# Genomics, molecular imaging, bioinformatics, and bio-nano-info integration are synergistic components of translational medicine and personalized healthcare research

**DOI:** 10.1186/1471-2164-9-S2-I1

**Published:** 2008-09-16

**Authors:** Jack Y Yang, Mary Qu Yang, Hamid R Arabnia, Youping Deng

**Affiliations:** 1 Harvard Medical School, Harvard University, P.O.Box 400888, Cambridge, Massachusetts 02115 USA; 2 National Human Genome Research Institute, National Institutes of Health, Bethesda, MD 20852 USA; 3 Department of Computer Science, University of Georgia, Athens, Georgia, 30602, USA; 4 Department of Biological Science, University of Southern Mississippi, Hattiesburg, 39406, USA

## Abstract

Supported by National Science Foundation (NSF), International Society of Intelligent Biological Medicine (ISIBM), International Journal of Computational Biology and Drug Design and International Journal of Functional Informatics and Personalized Medicine, IEEE 7th Bioinformatics and Bioengineering attracted more than 600 papers and 500 researchers and medical doctors. It was the only synergistic inter/multidisciplinary IEEE conference with 24 Keynote Lectures, 7 Tutorials, 5 Cutting-Edge Research Workshops and 32 Scientific Sessions including 11 Special Research Interest Sessions that were designed dynamically at Harvard in response to the current research trends and advances. The committee was very grateful for the IEEE Plenary Keynote Lectures given by: Dr. A. Keith Dunker (Indiana), Dr. Jun Liu (Harvard), Dr. Brian Athey (Michigan), Dr. Mark Borodovsky (Georgia Tech and President of ISIBM), Dr. Hamid Arabnia (Georgia and Vice-President of ISIBM), Dr. Ruzena Bajcsy (Berkeley and Member of United States National Academy of Engineering and Member of United States Institute of Medicine of the National Academies), Dr. Mary Yang (United States National Institutes of Health and Oak Ridge, DOE), Dr. Chih-Ming Ho (UCLA and Member of United States National Academy of Engineering and Academician of Academia Sinica), Dr. Andy Baxevanis (United States National Institutes of Health), Dr. Arif Ghafoor (Purdue), Dr. John Quackenbush (Harvard), Dr. Eric Jakobsson (UIUC), Dr. Vladimir Uversky (Indiana), Dr.  Laura Elnitski (United States National Institutes of Health) and other world-class scientific leaders. The Harvard meeting was a large academic event 100% full-sponsored by IEEE financially and academically. After a rigorous peer-review process, the committee selected 27 high-quality research papers from 600 submissions. The committee is grateful for contributions from keynote speakers Dr. Russ Altman (IEEE BIBM conference keynote lecturer on combining simulation and machine learning to recognize function in 4D), Dr. Mary Qu Yang (IEEE BIBM workshop keynote lecturer on new initiatives of detecting microscopic disease using machine learning and molecular biology,  http://ieeexplore.ieee.org/servlet/opac?punumber=4425386) and Dr. Jack Y.  Yang (IEEE BIBM workshop keynote lecturer on data mining and knowledge discovery in translational medicine) from the first IEEE Computer Society BioInformatics and BioMedicine (IEEE BIBM) international conference and workshops, November 2-4, 2007, Silicon Valley, California, USA.

## Introduction

Advent of high-throughput sequencing and advanced molecular imaging technologies marked the beginning of a new era for modern translational and personalized medicine. The impact of sequencing human and comparative genomes along with modern digital images in hand has also created the fast development of high performance computing and bioinformatics research, which will obviously have a profound effect on how biomedical research will be conducted toward the improvement of human health and prolonging of human life in the future. Supported by the United States National Science Foundation (NSF), the International Society of Intelligent Biological Medicine (ISIBM, http://www.ISIBM.org), the ISIBM's official journals: the International Journal of Computational Biology and Drug Design (IJCBDD, http://www.inderscience.com/IJCBDD), and the International Journal of Functional Informatics and Personalized Medicine (IJFIPM, http://www.inderscience.com/IJFIPM),  the IEEE 7th Bioinformatics and Bioengineering at Harvard Medical School was designed to touch future by promoting today's synergistic cutting edge research and education.  It was the only synergistic inter/multidisciplinary IEEE flagship conference in the field fully sponsored (100%) by the Institute of Electrical and Electronics Engineers  (IEEE) both financially and academically.    IEEE 7th Bioinformatics and Bioengineering received more than 600 research papers and attracted 500 scientists, engineers, medical doctors, faculty and students world-wide gathered at the conference center of Harvard Medical School during October 14–17, 2007 in Boston, Massachusetts USA.  All 600 submitted papers were carefully reviewed and evaluated by the program and scientific review committee consisting 273 professors, medical doctors and scientists. The 273 professors, medical doctors and scientists spent great amount of time and efforts in reviewing all submitted papers, tutorials, special interest research and workshop proposals. After the rigorous peer-review process, 27 high-quality research papers were selected from the 7th and 6th IEEE Bioinformatics and Bioengineering, keynote lectures and invited papers from world leading scientists for this BMC Genomics issue. The acceptances of the 27 papers out of 600 papers were based on peer-reviews. The committee is grateful for the contributions from the keynote speakers Dr. Russ B. Altman (IEEE BIBM conference keynote lecturer on combining simulation and machine learning to recognize function in 4D), Dr. Mary Yang (IEEE BIBM workshop keynote lecturer on New initiatives of detecting microscopic disease using machine learning and molecular biology,  http://ieeexplore.ieee.org/servlet/opac?punumber=4425386) and Dr. Jack Yang (IEEE BIBM workshop keynote lecturer on data mining and knowledge discovery in translational medicine) from the first IEEE Computer Society BioInformatics and BioMedicine (IEEE BIBM) international conference and workshops, November 2-4, 2007, Silicon Valley, California, USA.    The IEEE conference at Harvard has provided a forum for researchers and medical doctors presenting their latest work, sharing and exchanging research ideas, stimulating the potential collaboration and promoting the inter/multi-disciplinary research and education. The 27 cutting-edge research papers presented in this BMC Genomics issue addressed a wide range of research topics in gene regulation, gene network, gene structure prediction, DNA microarray data and ChIP-on-Chip data analysis, protein disorder, protein structure and function, disease prediction, genomics and bioinformatics tools. This BMC Genomics issue resonates the theme of the Harvard meeting: Genomics, Molecular Imaging, Bioinformatics, and Bio-Nano-Info Integration are Synergistic Components of Translational Medicine and Personalized Healthcare.  

## Scientific themes

This BMC genomics supplement consists of 27 research papers. Those papers can be grouped into following categories based on their contents.

### Protein structure and function prediction

More and more experimental evidences showed that many protein functions depend on the unstructured rather than structured state. Despite extensive data on many important examples, including disease-associated proteins, the importance of disorder for protein function has been largely ignored. As a pioneer in this research field, Dunker's group [1] reviewed the key discoveries and to weave these discoveries together to support novel approaches for understanding sequence-function relationships. They demonstrated that disorder prediction has been important for showing that the relatively few experimentally characterized examples are members of a very large collection of related disordered proteins that are wide spread over all three domains of life.

It is challenge to annotate the function of new proteins lacking of structure and sequence similarity with the known proteins for the traditional approaches. Altman's group [2] developed a novel framework, called FEATURE, to model and recognize of functional sites in macromolecular structures. FEATURE robustly models molecular functions without relying on significant sequence or fold similarity.

Protein interactions, which are mediated by domains, are important for most cellular functions. Ren et al. [3] systematically investigated the evolutionary conservation of SLiMs recognized by SH2, SH3 and Ser/Thr Kinase domains in both ordered and disordered protein regions. They found SLiMs recognized by each domain were more conserved in disordered regions as compared to structured regions of proteins. Their work could significantly contribute to protein-protein interaction study. Yang et al. [4] presented a machine learning approach to identify unstructured proteins. They demonstrated that IUP predictor is viable alternative software for prediction.

### Gene regulation and network

Bidirectional promoters were abundance in human genome and essential for many tumor suppressor genes. The functional mechanisms underlying the activation of bidirectional promoters are currently uncharacterized. Yang et al. [5] analyzed core promoter elements distribution in bidirectional promoters. They demonstrated that bidirectional promoters utilize a variety of core promoter elements to initiate transcription. CpG-islands dominate the regulatory landscape of this group of promoters.

Chen et al [6] developed a Network-Identifier method untilizing a thermodynamic model to infer regulatory relationships from multiple time course gene expression datasets. Applying Network-Identifier to five datasets of differentiating embryonic stem cells, they constructed a gene regulatory network among 87 transcription regulator genes.

Feng et al [7] presented a mixture model-based analysis for identifying differences in the distribution of RNA polymerase II from ChIP-seq in transcribed regions. They utilized a *Poisson *mixture model to identify gene targets with differential Pol II binding activities in breast cancer MCF7 cell line under various conditions. Their work provide a computational and statistical approaches to identify biological signals from ChIP-seq data.

### Gene structure and function

Wu [8] implemented a statistical approach that combined with evolutionary conservation information in gene prediction. Compared to results from GENSCAN and TWINSCAN, the author showed that this approach largely improved the specificity of exon prediction. They proposed the approach could serve as a complementary method to refine many existing approaches.

It is essential to align ontologies from different parties due to a great amount of variety of ontologies constructed under the auspices of the Open Biomedical Ontologies (OBO). Huang et al. [9] designed a new ontology alignment algorithm utilizing artificial neural network approach to learn and adjust the weights that associated with different semantic aspects.

### Comparative genomics

When transpositions are dominant, it turns to be very difficult to analyze genome rearrangement using existing approaches. Yue et al [10] extended GRAPPA, one of the most accurate methods for rearrangement phylogeny, to handle transpositions. Their test results showed that the extension approach is very accurate in terms of ancestor genome inference and phylogenetic reconstruction.

Array-based comparative genomic hybridization (array CGH) is a highly efficient technique that can simultaneously measure DNA copy number at hundreds or thousands of loci. Huang et al [11] developed Stationary Wavelet Packet to smooth the array CGH data. They demonstrated the effectiveness of their approach through theoretical and experimental exploration of a set of array CGH data. Their comparison results suggested their approach outperformed the previous methods.

### Gene expression and microarray data

There are two papers described their novel techniques of improving the quality of the microarray data. Yang et al. [12] exploited various data processing techniques to reduce the amount of inconsistence results in microarray experiments when genomic DNA was used as common reference. They found data quality was significantly improved by imposing the constraint of minimal number of replicates, logarithmic transformation and random error analyses. Mehdi et al. [13] implement interwoven design to improve microarray experiments to ensure that the resulting data enables robust statistical analysis. They developed an online web application that allows users to find optimal loop designs for two-color microarray experiments.

The clinical application of microarray data is been limited due to requirement to access detailed patient records. Harris et al. [14] presented the Genetic Learning Across Datasets concept (GLAD), a new Semi-Supervised Learning (SSL) method for combining independent annotated datasets and unannotated datasets with the aim of identifying more robust sample classifiers.

Gene expression data extracted from microarray experiments could generate high-dimensional feature space for discriminating between different classes when thousands of genes are measured simultaneously. Yana et al [15] investigated the feature selection methods that evaluate the "informativeness" of a set of genes. They demonstrated that including gene-gene interactions have better classification power in gene expression analysis. Li et al [16] presented a novel framework to select feature subsets from all the newly extracted components from PCA and PLS. They demonstrated that their feature selection scheme improved generalization performance of classifier according to the evaluation on several typical datasets. Zhang et al. [17] developed a two-stage selection algorithm by combining ReliefF and mRMR. Comparing to ReliefF, mRMR and other feature selection methods using two classifiers as SVM and Naive Bayes on seven different datasets, they showed that mRMR-ReliefF gene selection algorithm is very effective.

Xu et al [18] developed a novel approach combining both supervised learning with unsupervised learning techniques to generate discriminative gene clusters. Their experiments on both simulated and real datasets exhibited that their method could produce a series of robust gene clusters with good classification performance compared with existing approaches. Bryan et al [19] demonstrated that how the bicluster analysis may be extended into a 'semi-supervised' ORF annotation approach referred to as BALBOA. They showed the methods could improve upon supervised approaches and shed new light on the functions of unclassified ORFs and their co-regulation.

### Genomics and bioinformatics in disease study

Goh et al. [20] examined the usefulness of intrinsic disorder predictions for studying the viral proteins. They constructed a set of biocomputing tools that include relational database design and utilization of disorder prediction algorithms. Their exercise provides an example showing how the combined use of intrinsic disorder predictions and relational databases provides an improved understanding of the functional and structural behaviour of viral proteins.

Sudden death syndrome of soybean is an ec onomically important disease. Yuan et al [21] reported their analyses of microarrays measuring transcript in whole plants after *A. thaliana *cv 'Columbia' was challenged with fungal pathogen *F. virguliforme*. They found significant variations in transcript abundances which leaded to identification of a putative resistance pathway involved in responding to the pathogen infection in *A. thaliana*.

Yang et al. [22] investigated the cell killing effects on adjuvant RT and found that radio-sensitivity is actually not a monotonic function of volume as it was believed before. They presented detailed analysis and explanation to justify the statement and proposed an equivalent radio-sensitivity model. They conclude that radio sensitivity is a sophisticated function over tumor volumes.

Hewett et al. [23] investigated the possibility of making tumor classification more informative by using a method for classification ranking. They applied Multi Dimensional Ranker (MDR) on Microarray data of 11 different types and subtypes of cancer. They found that using the classification rankings from MDR could achieved effective tumor classifications from cancer gene expression data.

When the prostate cancer cell transfer from androgen-dependent to androgen-independent, androgen ablation, the most commonly-used therapy for progressive prostate cancer, is ineffective. Wang et al [24] developed a model-based computational approach to identify transcription factors and microRNAs influencing the progression of androgen-dependent prostate cancer to androgen-independent prostate cancer. This result suggested that the capability of transcription factors to initiate transcription and microRNAs to facilitate mRNA degradation are both decreased in androgen-independent prostate cancer.

Wu et al. [25] developed stringent statistical analysis approach to reduce type I and type II error and described its application in Type 1 diabetes research. They showed that stringent statistical analysis, combined with hierarchical clustering and pathway analysis may offer deeper insight into the biological processes reflected from a set of expression array data.

### Bio-informatics tool

Extracting information from batch BLAST can be consuming, insufficient, and inaccurate for large dataset. Mehdi et al. [26] developed a java application called Batch Blast Extractor to extract information from blast output. The application generates a tab delimited text file that can be easily imported into any statistical package such as Excel or SPSS for further analysis. The software is open access, free available to public.

Lieutaud et al. [27] developed a web metaserver called MeDor. MeDor provides a HCA plot and runs a secondary structure prediction, a prediction of signal peptides and transmembrane regions and a set of disorder predictions. This free available software tool could offer fast, simultaneous analysis of a query sequence by multiple predictors and provides a graphical interface with a unified view of the outputs.

## Promoting emerging cutting-edge research fields

Genomics, bioinformatics and personalized medicine are upcoming emerging fields, and the resonance and synergy of these fields are enormous, which will have profound influence on the advances of science and medicine. In cooperation with International Society of Intelligent Biological Medicine http://www.isibm.org, International Journal of Computational Biology and Drug Design (IJCBDD), International Journal of Functional Informatics and Personalized Medicine (IJFIPM) and World Congress on Computer Science, Computer Engineering and Applied Computing http://www.world-academy-of-science.org, and built on the great success of the International Conference on Bioinformatics and Computational Biology (Biocomp), the IEEE 7^th ^Bioinformatics and Bioengineering was unique because of a number of reasons: The committee emphasized the promotion of inter/multidisciplinary research and education in accepting papers and designing of the programs. One of the most important contributions of this conference based on the great success of the 2007 International Conference on Bioinformatics and Computational Biology (Biocomp 2007) was that it created interaction and research collaboration in inter/multidisciplinary research amongst the Biophysical Sciences, Medical Sciences, Agricultural Sciences, Computer Science and Engineering by providing a productive and rich platform. The location of the conference (Harvard Medical School) was strategically chosen to attract the participation of renowned researchers who work in Cambridge – Boston, Massachusetts area which is an international biomedical research center (radius of 50 miles). Collaborative inter/multidisciplinary research will help researchers from all disciplines involved, to reach the summit in their sub-areas of the Bioinformatics and Bioengineering related projects. By doing so, both Worldcomp in Las Vegas and the IEEE 7^th ^Bioinformatics and Bioengineering at Harvard Medical School have achieved the goal of addressing challenges in the emerging contemporary Bioinformatics and Bioengineering fields that would not further exist "as is" in separate silos as isolated fields, but instead spanning a wide spectrum of knowledge and research focusing on the synergy of these two fields to solve important but difficult biological and medical problems. It is strongly believed that people from various research backgrounds would extend their knowledge scopes and conceive some novel research ideas inspired by extensive inter/multidisciplinary discussions and collaborations. Not many conferences would have such multilateral cooperative efforts, with the help from ISIBM and the Worldcomp, the IEEE 7^th ^Bioinformatics and Bioengineering was so unique to help to pass the hurdles and break the invisible academic barriers between these two different but both very important disciplines. Most updated cutting-edge technologies and breaking though ideas had brought into the conference at Harvard Medical School in the keynote and tutorial lectures, and the open discussions and scientific exchanges among attendees, which inspired innovations, novel ideas and scientific discoveries. The IEEE 7^th ^Bioinformatics and Bioengineering provided such unique infrastructure and platform to promote interdisciplinary and multidisciplinary research and education.

The conference brought together top researchers from the United States and around the world to exchange research results and address open issues in all aspects of bioinformatics and bioengineering. The IEEE conference hosted a number of cutting-edge research workshops and special interest research sessions in collaboration with leading scientists from the National Human Genome Research Institute (NHGRI) and National Cancer Institute (NCI), National Institutes of Health (NIH), U.S. Department of Health and Human Services, Oak Ridge National Laboratory, U.S. Department of Energy, Indiana University, Purdue University, Georgia Institute of Technology, Southern Illinois University, University of Illinois, University of Michigan, University of California, University of Massachusetts, Harvard Medical School, Harvard University and a number of other national laboratories and major research universities.

Built on the great success of Biocomp – Worldcomp, the aim of the IEEE 7^th ^Bioinformatics and Bioengineering was to assemble a spectrum of affiliated research workshops, distinguished keynote and tutorial lectures and special interest research sessions into a coordinated research meeting. Due to the broad knowledge and scopes that IEEE 7^th ^Bioinformatics and Bioengineering encompassed, the conference received more than 600 high-quality research papers. The traditional IEEE Computer Society 6^th ^Bioinformatics and Bioengineering received 100+ high-quality papers. A significant expansion has been achieved and the acceptance rate for regular research papers was less than 12%. The papers covered broad range of research fields including genomics, bioinformatics and bioengineering that created the theme of the IEEE flagship conference: genomics, molecular imaging, bioinformatics, and bio-nano-info integration are synergistic components of translational and personalized medicine research.

Major organizers of the IEEE 7th Bioinformatics and Bioengineering who hold formal degrees in the fields of both engineering and biophysical/biomedical sciences with many years of research, teaching and engineering practice experiences realized the power and needs of synergistic engineering and biomedical research based on their professional experience. They and all other organizers are dedicated to promote such inter/multidisciplinary research and education. Therefore, the IEEE 7th Bioinformatics and Bioengineering has been designed to be responsive to the need for synergistic inter/multidisciplinary research and education. For that reason, it is the only meeting whose components are also defined dynamically in response to specific needs of largest number of keynote lectures, cutting-edge research tutorial lectures, special interest research workshops and special sessions with academic supports and contributions from leading scientists at NIH, national laboratories and research universities. Each proposal and nomination has been reviewed and voted by the IEEE 7th Bioinformatics and Bioengineering committee to ensure that participants would be benefited significantly from the academic event. We are very grateful for the IEEE Bioinformatics and Bioengineering Plenary Keynote Lectures given by:

1. Dr. A. Keith Dunker (Indiana University and Purdue University),

2. Dr. Jun S. Liu (Harvard University and Massachusetts Institute of Technology),

3. Dr. Andy Baxevanis (National Human Genome Research Institute, National Institutes of Health, United States Department of Health and Human Services),

4. Dr. Mark Borodovsky (International Society of Intelligent Biological Medicine and Georgia Institute of Technology),

5. Dr. Brian D. Athey (University Michigan and the National Center for Integrative Biomedical Informatics),

6. Dr. Hamid R. Arabnia (International Society of Intelligent Biological Medicine and University of Georgia)

7. Dr. Ruzena Bajcsy (University of California at Berkeley and Member of United States National Academy of Engineering and, Member of United States Institute of Medicine of the National Academies).

8. Dr. Ferenc A. Jolesz (Harvard University and Member of United States Institute of Medicine of the National Academies).

9. Dr. Mary Qu Yang (National Human Genome Research Institute, National Institutes of Health, U.S. Department of Health and Human Services and Oak Ridge, DOE),

10. Dr. Chih-Ming Ho (University of California at Los Angles and Member of United States National Academy of Engineering and Academician of Academia Sinica).

11. Dr. John Quackenbush (Harvard School of Public Health & Dana-Farber Cancer Institute),

12. Dr. Arif Ghafoor (Purdue University, West Lafayette),

13. Dr. Laura L. Elnitski (National Human Genome Research Institute, National Institutes of Health (NIH), U. S. Department of Health and Human Services),

14. Dr. Eric Jakobsson (University of Illinois at Urbana-Champaign and the National Center for Design of Biomimetic Nanoconductors)

15. Dr. Tony Xiaohua Hu (Drexel University and the IEEE Computer Society),

16. Dr. Patrick S. P. Wang (Northeastern University and Massachusetts Institute of Technology),

17. Dr. Steven E. Seltzer (Harvard Medical School & Brigham & Women's Hospital),

18. Dr. Yi Pan (Georgia State University),

19. Dr. Jay Steve Loeffler (Harvard Medical School and Massachusetts General Hospital),

20. Dr. Yuehui Chen (University of Jinan)

21. Dr. Linda Molnar (National Cancer Institute, National Institutes of Health (NIH), United States Department of Health and Human Services),

22. Dr. Vladimir N. Uversky (Indiana University School of Medicine),

23. Dr. Gary Gottlieb (Harvard Medical School and Brigham & Women's Hospital),

24. Dr. Larry O. Hall (University of South Florida and the IEEE/SMC).

And the Advanced Cutting-Edge Research and Education Tutorial Lectures given by:

1. Dr. David Lightfoot (Southern Illinois University, Carbondale),

2. Dr. Igor B. Zhulin (Oak Ridge National Laboratory, DOE and University of Tennessee),

3. Dr. May D. Wang (Georgia Institute of Technology and Emory University),

4. Dr. Yehuda Braiman (Oak Ridge National Laboratory, U.S. Department of Energy),

5. Dr. Craig A. Stewart (Indiana University, Bloomington),

6. Dr. Tian Zheng (Columbia University),

7. Dr. Michelle M. Zhu (International Society of Intelligent Biological Medicine, and Oak Ridge National Laboratory and the Southern Illinois University)

Obviously, the conference would not have achieved such a great success without the hard work and voluntary efforts by many contributors. Organizing such a major academic event in the fields is not possible without contributions from members of program and scientific review committee. Thanks must be given to them for their professionalisms. We must express our sincere gratitude to Mary Qu Yang, Vladimir N. Uversky, Yunlong Liu and the program and scientific review committee members for their high-quality timely evaluation of more than 600 full-length regular research papers, as well as an additional large set of poster submissions. We must extend our sincere thanks to all chairs, organizers and committee members (names and affiliations are listed below) for their dedications and professional services. In particular, Hamid R. Arabnia, Walker Land, Jr., Homayoun Valafar and the award committee members dedicated themselves to the unbiased evaluation and selection of the best papers, and the NSF student travel fellowships; Yanqing Zhang and Michelle M. Zhu managed the paper submission system and handled various important organizing and academic affairs; Youping Deng handled not only registration and finance but also reviewed a large number of papers; Jonathan Jesneck and Pengyu Hong helped local arrangements; Qingzhong Liu, Mehdi Pirooznia, Zejin Jason Ding, and Bingxin Shen maintained the website. Walker Land, Jr., Guo-Zheng Li, Hamid R. Arabnia, Youping Deng, Michelle M. Zhu and Jack Y. Yang helped Scientific Review Committee Co-Chairs in the overall systematic evalutions of all reviewers' comments and ranks. Michelle M. Zhu, Heng Huang, Nikolaos G. Bourbakis and Jun Ni helped in the overall organization of the conference and Michelle M. Zhu arranged the scientific presentations and program schedules; Jack Y. Yang initiated the organization of the IEEE 7th Bioinformatics and Bioengineering and guided the committees as well as managed the overall infrastructure of the IEEE conference. Mary Qu Yang and Jack Y. Yang initiated not only the journal issues but also the NSF proposals that provided funds to support students' travel; this is the first time that IEEE Bioinformatics and Bioengineering is now not only offering leading scientific journal issues such as *BMC Genomics *(impact factor: 4.03) and *International Journal of Computational Biology and Drug Design*, but also receiving grants and support from the National Science Foundation. Mary Qu Yang and Jack Y. Yang reformed the traditional IEEE Bioinformatics and Bioengineering with new components that are defined dynamically in response to specific needs of inter/multidisciplinary cutting-edge research and education, therefore, Mary Qu Yang and Jack Y. Yang initiated and arranged the organization of cutting-edge research workshops, tutorial lectures, special sessions and poster presentations in addition to the traditional keynote lectures. Mary Qu Yang and Jack Y. Yang took the initiatives and invested significant efforts to expand the size of IEEE Bioinformatics and Bioengineering from traditional 100–200 papers each year for past 6 years to more than 600 full-length regular research paper submissions this year.

The theme of the IEEE 7^th ^Bioinformatics and Bioengineering at Harvard Medical School was to promote the genomics, molecular imaging, bioinformatics, and bio-nano-info integration research that are important components of translational and personalized medicine. Intellectual Merits include:

(1) Maximize learning outcomes (a lot of internationally recognized experts delivered keynotes and tutorials with large audience. The great learning outcomes have achieved, and

(2) Maximize research outcomes (because students and young investigators are major future research experts and next-generation scientists to develop new techniques in bioinformatics, genomics and bioengineering, now it is necessary to provide them with the best chances to improve themselves. So the more students and scientists attended, the more benefits in research knowledge they would obtain. The conference has achieved above goal using the model of World Congress on Computer Science, Computer Engineering and Applied Computing.

Broader impacts include

(1) Benefits in education: students and researchers who were really needed helps do have chances to attend IEEE 7^th ^Bioinformatics and Bioengineering to meet experts and listen to research presentations with a travel fellowship program, and

(2) Benefits in research: students and scientists had opportunities to discuss research ideas with experts and have future collaborative projects with other researchers.

### The NSF student travel fellowship and best paper awards

To increase participation of underrepresented groups such as minorities, women, and disabled individuals, Drs. Jack Y. Yang, Yanqing Zhang, and Youping Deng submitted a grant proposal to the United States National Science Foundation, which led to a successfully funding of IEEE 7^th ^Bioinformatics and Bioengineering by the NSF. A committee chaired by Prof. Hamid R. Arabnia, and Co-Chaired by Prof. Walker Land, Jr., Prof. Homayoun Valafar, Prof. Yunlong Liu, Prof. Michelle M. Zhu, Prof. Jonathan Jesneck, Prof. Yuehui Chen, Prof. Yufang Jin, Prof. Alex Zelikovsky, Prof. Chung-Kuan Cheng and Prof. Anu Bourgeois was formed to select the NSF student travel fellowships, best papers and best posters. IEEE Bioinformatics and Bioengineering Best Paper Awards were conferred to the authors of (1) the best research papers (2) the best application papers (3) the best inter/multidisciplinary papers (4) the best student papers. Foundational and original results were considered for the best research paper awards; application-oriented submissions were considered for the best application paper awards; original research papers that significantly promote synergistic inter/multidisciplinary research were considered for the best inter/multidisciplinary papers and papers first authored by students (graduate or undergraduate full-time students) were considered for the best student paper awards. The winners determined by the committee are listed below:

NSF student travel fellowship awards

Alessandro Abate, University of California at Berkeley

Benard Chen, Georgia State University

Stephanie Jimenez Irausquin, University of South Carolina

Vidya Kamath, University of South Florida

Majid Masso, George Mason University

John Jiazheng Yuan, Southern Illinois University

Arun Rawat, University of Southern Mississippi

Qiong Chen, Georgia State University

Best Research – Best Paper Awards:

First Runner-Up Best Student Research

Bernard Chen, Stephen Pellicer, Phang C. Tai, Robert Harrison and Yi Pan.

Super Granular SVM Feature Elimination (Super GSVM-FE) Model for Protein Sequence Motif Information Extraction

Georgia State University

Best Student Research

Alessandro Abate, Yu Bai, Nathalie Sznajder, Carolyn Talcott and Ashish Tiwari.

Quantitative and Probabilistic Modeling in Pathway Logic

University of California at Berkeley Stanford University

First Runner-Up Best Application Research

Stephanie Jimenez Irausquin and Liangjiang Wang.

A Machine Learning Approach for Prediction of Lipid-Interacting Residues in Amino Acid Sequences.

Clemson University

University of South Carolina

Best Application Research

Jeffrey Tilson, Gloria Rendon, Mao-Feng Ger and Eric Jakobsson.

MotifNetwork: A Grid-enabled Workflow for High-throughput Domain Analysis of Biological Sequences

University of Illinois at Urbana-Champaign

First Runner-Up Best Inter/Multidisciplinary Research Shibin Qiu and Terran Lane.

The RNA String Kernel for siRNA Efficacy Prediction.

University of New Mexico

Best Inter/Multidisciplinary Research

Guo-Zheng Li, Xue-Qiang Zeng, Jack Y. Yang and Mary Qu Yang

Partial Least Squares Based Dimension Reduction with Gene Selection for Tumor Classification.

Shanghai University

Harvard University

National Institutes of Health (NIH).

First Runner-Up Best Original Research

Yunfeng Yang, Mengxia (Michelle) Zhu, Liyou Wu and Jizhong Zhou.

Biostatistical Considerations of the Use of Genomic DNA Reference in Microarrays.

Oak Ridge National Laboratory, United States Department of Energy.

Southern Illinois University.

Best Original Research

Keith Dunker, Christopher J. Oldfield, Jingwei Meng, Pedro Romero, Jack Y. Yang, Zoran Obradovic and Vladimir N. Uversky.

Intrinsically Disordered Proteins: An Update.

Indiana University

Indiana University Purdue University

The IEEE 7th Bioinformatics and Bioengineering as a large IEEE flagship international conference with 7 years of tradition and leading reputation provided an important platform at the conference center of Harvard Medical School for scientific discussions and collaborations. Because the IEEE 7^th ^Bioinformatics and Bioengineering received more than 600 papers in more diverse fields, the committee had decided to review and rank the papers in different categories in accordance to the enormous expansion of the conference. For example, the Best Inter/Multidisciplinary research category was newly created by the committee. Comparably, the IEEE 6^th ^Bioinformatics and Bioengineering was a traditional IEEE Computer Society flagship conference held in Washington, DC – Arlington Virginia on October 15–18, 2006, the IEEE Computer Society conference received more than 100 high-quality papers in more traditional bioinformatics field. The IEEE 6th Bioinformatics and Bioengineering committee chaired by Prof. Nik Bourbakis, Prof. Michael Raymer and Prof. George Karypis determined the following Outstanding Achievement Awards:

Best Original Research Paper Award (also Best Student Paper Award),

Sridhar Ramachandran, Travis Doom, Michael L. Raymer and Dan Krane

Parsimony based Approach to Test the Evolving Master Gene Hypothesis for Human Alu Repeats

Wright State University, Dayton, Ohio, USA

Best Software Tool and Application Research Paper Award

Mary Qu Yang and Jack Y. Yang

IUP : Intrinsic Unstructured Protein Prediction – A Software Tool for Analyzing Polypeptide Structures

National Human Genome Research Institute, National Institutes of Health (NIH), U.S. Department of Health of Human Services.

Harvard Medical School, Harvard University

Educational Service Award

Cathy H. Wu

Genomic and Proteomic Bioinformatics at the Protein Information Resource.

Georgetown Medical Center, Georgetown University

## The role of inter/multidisciplinary research and education

Bioinformatics, genomics and bioengineering play fundamental roles in our understanding and designs of biological systems and therapy medicine at all levels of organization, from molecular biology, life sciences to engineering and computer sciences. Bioinformatics is a "bourgeoning out" field that studies the development of algorithms, computational and statistical techniques, and theories to solve formal and practical biomedical problems. It also refers to hypothesis-driven investigations of specific biological problems using computer simulations that carry out with experimental or simulated data, with the primary goal of discovery and the advancement of biological knowledge. Bioinformatics deals with the information and hypotheses. It is a technique-driven research that utilizes many engineering and computer science methods. Bioinformatics focus on the developments of mathematical and computational tools to extract useful information from data produced by high-throughput biological techniques such as genome sequencing, protein sequences, gene regulation, gene networks, ChIP-on-chip and DNA microarrays data as well as mass spectrometry and MRI. Bioinformatics broadly includes systems biology and computational biology.

Bioengineering is a broad-based engineering discipline that studies engineering biological processes involving product design, sustainability and analysis of biological systems. Bioengineering deals with all the biological, medical, pharmaceutical the agricultural fields using engineering methods and approaches. Bioengineering thus deals with living organism and biological products and broadly includes food engineering and biotechnology. It is an engineering field because it deals with practical designs to create usable tangible products. In general, bioengineering attempts to solve the following biomedical problems: 1) mimic biological systems in order to create products; 2) modify and control biological systems so that they can replace, augment, or sustain chemical and mechanical processes; 3) design and developments of medical devices and medical physics technologies.

In order to understand the life, studies of living organisms must be carried out at multiple levels. Bioinformatics deals with problems more at the microscopic level such as genomic, proteomic and molecular biology data analysis, while bioengineering focuses more on macroscopic level such as tissue engineering, cell engineering, medical image analysis and biological and medical devices. However, bioinformatics and bioengineering often exploit the same or similar engineering and computational approaches to solve practical or potentially practical biomedical problems, despite of their different focuses and different studying objects. Because any life is an integrated living organism, studies and research on living organisms must be carried out at global level in order understand the mechanism of living organisms and in order to design effective therapy methods to treat human diseases and promote human health. Up until now research on these two important engineering related fields have been carried out in separate silos, but the synergy of the research on both bioinformatics and bioengineering will prove beneficial.

It is evidently that bioinformatics, genomics and bioengineering all study living organisms from different angles and levels with different focuses but often with same or similar engineering and computer science methods. Since any living organisms are integrated life form and baring this in mind, understanding the mechanisms of living organism and breaking through advances in biological, medical and agricultural sciences etc. require comprehensive studies and research of living organisms at multiple levels and require the combination and integration of knowledge acquired from multiple levels. Using World Congress on Computer Science, Computer Engineering and Applied Computing (Wordcomp) as a model, the IEEE 7^th ^Bioinformatics and Bioengineering was aimed to bridge the gaps of biomedical researches at different levels of studies on living organisms and fosters interdisciplinary and multidisciplinary research and education.

High throughput sequencing and modern imaging technologies foster the development of translational and personalized medicine which use of information and data from genotype, patient's phenotype, level of gene expression and protein interaction to discover biological mechanism, stratify disease, select a medication, plan a therapy, or initiate a preventative measure that is particularly suited to that patient at the time of administration. In addition to genetic information, other factors, including protein disorder, protein aggregation, protein misfolding, medical imaging, and clinical information also play important roles. The 2007 International Conference on Bioinformatics and Computational Biology (Biocomp) was designed in response to the fast development of above inter/multidisciplinary fields [28], 25 high-quality papers [29-53] were selected for a *BMC Genomics *supplementary issue. Built on the great success of the Biocomp, the IEEE 7^th ^Bioinformatics and Bioengineering was dedicated to explore and develop such synergies to promote genomics, molecular imaging, bioinformatics, and bio-nano-info integration that are key components of translational and personalized medicine research, the 27 research papers in this *BMC Genomics *issue resonate above theme.

### The IEEE 7th Bioinformatics and Bioengineering Organization

Jack Y. Yang

General Chair and Coordinator of Committees on the IEEE 7th Bioinformatics and Bioengineering, Harvard University, Harvard Medical School, USA

Mary Qu Yang

Scientific Review, Advisory, and Steering Committee Chair

National Human Genome Research Institute, National Institutes of Health (NIH), U.S. Department of Health and Human Services, and Oak Ridge, DOE, USA

Michelle M. Zhu

Program Chair

Secretary-General of the International *Society of Intelligent Biological Medicine*, Executive Managing Editor of *International Journal of Computational Biology and Drug Design*, Managing Editor of *International Journal of Functional Informatics and Personalised Medicine*, Oak Ridge National Laboratory, DOE and Southern Illinois University, USA

Hamid R. Arabnia

Program Co-Chair and Award Committee Chair, Vice-President of the *International Society of Intelligent Biological Medicine*, Head of Panel on Best Papers & Travel Support, University of Georgia, USA

Youping Deng

Program Co-Chair and Registration/Budget Committee Chair, Bioinformatics Associate Editor of the *BMC Research Notes *and Vice-President of the *International Society of Intelligent Biological Medicine*, University of Southern Mississippi, USA

Yanqing Zhang

Program Co-Chair and Bioinformatics Track Chair, Georgia State University, USA

George T. Y. Chen

Program Co-Chair and Bioengineering Track Chair, Harvard University, Harvard Medical School and Massachusetts General Hospital, USA

Lawrence O. Hall

President of IEEE/SMC, University of South Florida, USA

Zhi-Pei Liang

Co-Chair of Advisory Committee, IEEE/EMBS Vice-President for Conferences, University of Illinois at Urbana Champaign, USA

A. Keith Dunker

Chair of Workshop Proposal Committee, Indiana University, USA

Ying Xu, Co-Chair of Workshop Proposal Committee, University of Georgia and Oak Ridge National Laboratory, DOE, USA

Nikolaos G. Bourbakis, Chair of Founding Steering Committee, Wright State University, USA

Heng Huang, Organizing Chair, Deputy Secretary-General of the *International Society of Intelligent Biological Medicine*, Executive Managing Editor of the *International Journal of Functional Informatics and Personalised Medicine*, University of Texas at Arlington, USA

Organizers of Workshop on Bio-Nano-Info Integration for Personalized Medicine

May D. Wang, Georgia Institute of Technology and Emory University School of Medicine.

Linda Molnar, National Cancer Institute, NIH, U.S. Dept. of Health and Human Services, USA.

Eric Jakobsson, National Center for Design of Biomimetic Nanoconductors, and National Center for Supercomputing Applications, University of Illinois at Urbana-Champaign, USA

Organizer of Workshop on Progress toward Petascale Applications in Bioinformatics and Computational Biology

Craig A. Stewart and Malinda Lingwall, Indiana University, Bloomington, USA David Bader, Georgia Institute of Technology

Organizers of Workshop on Joint Research in the Southern Illinois University, University of Illinois, and Oak Ridge National Laboratory, U.S. Department of Energy, U.S.A.

Michelle M. Zhu and William P. Osborne, Southern Illinois University, USA

Laura L. Elnitski, National Human Genome Research Institute, NIH, U.S. Department of Health

Zhi-Pei Liang, IEEE/EMBS Vice-President for Conferences, University of Illinois, USA Igor B. Zhulin, University of Tennessee and Oak Ridge National Laboratory, DOE, USA Yehuda Braiman, Oak Ridge National Lab, DOE, USA

Organizers of IEEE 7th Bioinformatics and Bioengineering Special Workshop – The 4th Annual University of Massachusetts Bioinformatics Conference (folded into main conference)

Georges Grinstein and Ken Marx, University of Massachusetts Lowell, USA

Organizers of Pacific Bioinformatics and Biomedical Engineering Research Workshop (folded into main conference)

Ron Kikinis, Harvard University, Harvard Medical School and BWH, USA

Yuehui Chen, University of Jinan, China

J. Paul Robinson, Purdue University, West Lafayette, USA

Andrew H. Sung, New Mexico Tech., USA

Guo-Zheng Li, Shanghai University, China

Organizer of Special Session on Sequence Alignment and Phylogenetic Analysis. Mark Clement, Brigham Young University, USA

Organizers of Special Session on DNA Microarray Data Analysis.

Chris Ding, Lawrence Berkeley National Laboratory and University of California, USA

Heng Huang, University of Texas at Arlington, USA

Organizers of Special Session on Bio-Medical Soft Computing

Qingzhong Liu, Srinivas Mukkamala and Andrew H. Sung, New Mexico Tech., USA

Organizers of Special Session on Research in Bioinformatics, Neuroinformatics, and Systems Biology in East Asia.

Hyunsoo Kim, Georgia Institute of Technology, USA

Sun Kim, Indiana University, Bloomington, USA

Doheon Lee, Korea Advanced Institute of Science and Technology, Korea

Organizers of Special Session: Developing Algorithms for Solving Problems in Molecular Biology and Bio-Medicine.

Guo-Zheng Li, Shanghai University, China

Saurabh Sinha, University of Illinois at Urbana-Champaign, USA

Organizer of Special Session on High-throughput Data Analysis for Genomics and Proteomics.

Jean Gao, University of Texas at Arlington, USA.

Organizers of Special Session on Computational Intelligence in Medical Informatics.

Jianlin Cheng, University of Missouri, USA

Jijun Tang, University of South Carolina, USA

Organizers of Special Session on Bio-Complexity.

Matthias Dehmer, Max F. Perutz Laboratories, Vienna Bio Center, Austria

Frank Emmert-Streib, Washington University, USA

Organizers of Special Session on Evolutionary Systems Biology.

Xun Gu, Iowa State University, USA

Yufeng Wang, University of Texas at San Antonio, USA

Organizer of Special Session on Pattern Recognition and Gene Discovery in Molecular Genetics.

Sridhar Ramachandran, Indiana University, Southeast, USA

Organizer of Special Session on Machine Learning Methods in Structural/Functional Genomics.

Rui Kuang, University of Minnesota, USA

Organizers of Special Session on Biomedical Engineering for Handicapped Individuals

Nikolaos G. Bourbakis, Wright State University, USA

Jack Y. Yang, Harvard University, Harvard Medical School, USA

Inter/Multidisciplinary Chair

Mary Qu Yang, National Human Genome Research Institute, NIH, and Oak Ridge, DOE, USA

Co-Chairs of Publication Committee

Yuehui Chen, University of Jinan, China

Yufang Jin, University of Texas at San Antonio, USA

Alex Zelikovsky, Georgia State University, USA

Co-Chairs of Award Committee

Hamid R. Arabnia, University of Georgia, USA

Walker Land, Jr., Binghamton University, USA

Homayoun Valafar, University of South Carolina, Columbia, USA

Head of Panel on Best Papers and NSF Travel Fellowships

Hamid R. Arabnia, University of Georgia, USA

Co-Chairs of Tutorial Proposal Committee

Joydeep Ghosh, University of Texas at Austin, USA

Vasile Palade, Oxford University, United Kingdom

My Tra Thai, University of Florida, USA

Poster Chairs

Chung-Kuan Cheng, University of California at San Diego, USA

Jonathan Jesneck, Broad Institute of Massachusetts Institute of Technology and Harvard, USA

Michael L. Raymer, Wright State University, USA

Publicity Chairs

Okan K. Ersoy, Purdue University, West Lafayette, USA

Lihua Li, Hangzhou Dianzi University, China

Yingshu Li, Georgia State University, USA

Liqiang Zhang, Indiana University, South Bend USA

Web Chairs

Zejin Jason Ding, Georgia State University, USA

Qingzhong Liu, New Mexico Tech, USA

Mehdi Pirooznia, University of Southern Mississippi, USA

Bingxin Shen, Brookhaven National Lab and Stony Brook University, USA

Industry Chairs

Kevin Daimi, University of Detroit Mercy, USA

Deanne Taylor, Harvard School of Public Health, Harvard University, USA

Joe Zhou, Monash University, Australia and Hong Kong Polytechnic University, Hong Kong

International Chair (Visa Request Chair)

Anu Bourgeois, Georgia State University, USA

Local Chairs

Gil Alterovitz, Harvard Medical School and Massachusetts Institute of Technology, USA

Marek Ancukiewicz, Harvard Medical School and Massachusetts General Hospital, USA

Jonathan Jesneck, Harvard Medical School and Massachusetts Institute of Technology, USA

Pengyu Hong, Brandeis University and National Center of Behavioral Genomics, USA

Marco Ramoni, Harvard Medical School and Massachusetts Institute of Technology, USA

Program Vice-Chairs

Yuehui Chen, University of Jinan, USA

Jun Ni, University of Iowa USA

Vladimir N. Uversky, Indiana University School of Medicine, USA

Patrick S. Wang, Northeastern University, USA

Co-Chairs of Scientific Review Committee

Yunlong Liu, Indiana University Purdue University, USA

Vladimir Uversky, Indiana University School of Medicine, USA

Mary Qu Yang, National Human Genome Research Institute, NIH, & Oak Ridge, DOE. USA

Co-Chairs of Advisory Committee

Zhi-Pei Liang, IEEE/EMBS Vice-President for Conferences, University of Illinois, USA.

Mary Qu Yang, National Human Genome Research Institute, NIH, and Oak Ridge, DOE, USA

### Members of program and scientific review committee

1. Purang Abolmaesumi, Queen's University, Canada

2. Ajith Abraham, Chung-Ang University, South Korea

3. Osman Abul, TOBB University of Economics and Technology, Turkey

4. R. Acharya, Pennsylvania State University, USA

5. Reda Alhajj, University of Calgary, Canada

6. Gil Alterovitz, Massachusetts Institute of Technology and Harvard University, USA

7. Sergio A. Alvarez, Boston College, USA

8. Marek Ancukiewicz, Harvard University, Harvard Medical School and MGH, USA

9. Neculai Archip, Harvard University, USA

10. Babak Akhgar, Sheffield Hallam University, UK

11. Hisham Al-Mubaid, University of Houston – Clear Lake, USA

12. Hamid R. Arabnia, University of Georgia, USA

13. Brain D. Athey, University of Michigan, USA

14. Fred Azar, University of Pennsylvania, USA

15. David Bader, Georgia Tech, USA

16. Ruzena Bajcsy, University of California-Berkeley, USA

17. Chengpeng Bi, University of Missouri Kansas City & Children's Mercy Hospitals, USA

18. Emad Boctor, Johns Hopkins University, USA

19. Nazeih Botros, Southern Illinois University, Carbondale, USA

20. Nikolaos G. Bourbakis, Wright State University, USA

21. Anu Bourgeois, Georgia State University, USA

22. Yehuda Braiman, Oak Ridge National Lab, DOE, USA

23. Hong Cai, University of Texas at San Antonio, USA

24. Liming Cai, University of Georgia, USA

25. Rui Camacho, LIACC/FEUP University of Porto, Portugal

26. Ada Chen, Southern Illinois University, USA

27. Bin Chen, Duke University, USA

28. George T. Y. Chen, Harvard University, Harvard Medical School and MGH, USA

29. Jianer Chen, Texas A&M University, USA

30. Luonan Chen, Osaka Sangyo University, Japan

31. Yuehui Chen, University of Jinan, USA

32. Chao Cheng, University of Southern California, USA

33. Jianlin Cheng, University of Missouri, USA

34. Chung-Kuan, Cheng, University of California (UCSD), USA

35. Qiang Cheng, Wayne State University and Southern Illinois University, USA

36. Sheng Cheng, Shanghai Jiaotong University, China

37. Annie Chiang, Stanford University, USA

38. Sung-Bae Cho, Yonsei University, Korea

39. Albert C. S. Chung, Hong Kong University of Science and Technology, Hong Kong

40. Mark Clement, Brigham Young University, USA

41. Craig W. Codrington, Purdue University, USA

42. Jason Corso, University at Buffalo, USA

43. Jessica Crouch, Old Dominion University, USA

44. Kevin Daimi, University of Detroit Mercy, USA

45. Phuongan Dam, University of Georgia, USA

46. Karen Daniels, University of Massachusetts Lowell, USA

47. Matthias Dehmer, Vienna Bio Center, Austria

48. Youping Deng, University of Southern Mississippi, USA

49. Chris Ding, Lawrence Berkeley National Lab. – University of California, USA

50. Zejin Ding, Georgia State University, USA

51. Karin Dorman, Iowa State University, USA

52. Cheng Du, Chinese Academy of Science, China

53. Ye Duan, University of Missouri, USA

54. Zhong-Hui Duan, University of Akron, USA

55. Werner Dubitzky, University of Ulster, Coleraine, UK

56. A. Keith Dunker, Indiana University Purdue University, USA

57. Mark Ebbert, Brigham Young University, USA

58. Laura L. Elnitski, National Human Genome Research Institute, NIH, USA

59. Frank Emmert-Streib, University of Washington, USA

60. Okan K. Ersoy, Purdue University, West Lafayette, USA

61. Michael Folkert, Massachusetts Institute of Technology and Harvard University, USA

62. Jean Gao, University of Texas at Arlington, USA

63. Jane Geisler-Lee, Southern Illinois University, USA

64. Arif Ghafoor, Purdue University, West Lafayette, USA

65. Joydeep Ghosh, University of Texas at Austin, USA

66. Vicente Grau, University of Oxford, UK

67. Georges Grinstein, University of Massachusetts Lowell, USA

68. Jianying Gu, City University of New York, USA

69. Juntao Guo, University of Georgia, USA

70. Aili Han, Shandong University at Weihai, China

71. Cliff S. Han, Los Alamos National Laboratory, DOE. USA

72. Robert Harrison, Georgia State University, USA

73. Aristotelis Hatzioannou, the National Science Foundation of Greece, Greece

74. Haibo He, Stevens Institute of Technology, USA

75. Rattikorn Hewett, Texas Tech University, USA

76. Vasant Honavar, Iowa State University, USA

77. Pengyu Hong, Brandeis University and National Center for Behavior Genomics, USA

78. David Hsu, National University of Singapore, Singapore

79. Wen-Lian Hsu, Academia Sinica, Taiwan

80. Tony Xiaohua Hu, Drexel University, USA

81. Yuh-Jyh Hu, National Chiao Tung University, Taiwan

82. Heng Huang, University of Texas at Arlington, USA

83. Xudong Huang, Harvard University and Massachusetts Institute of Technology, USA

84. Yalou Huang, Naikai University, China

85. Eric Jakobsson, University of Illinois at Urbana-Champaign, USA

86. Jonathan Jesneck, Harvard University, Harvard Medical School and DFCI, USA

87. Tao Jiang, University of California-Riverside, USA

88. Tianzi Jiang, Chinese Academy of Science, China

89. Yuan Jiang, Nanjing University, China

90. Bo Jin, Medical University of South Carolina, USA

91. Yufang Jin, University of Texas at San Antonio, USA

92. Mehmed Kantardzic, University of Louisville, USA

93. George Karypis, University of Minnesota, USA

94. Paul Keall, Stanford University, USA

95. Arpad Kelemen, Niagara University, USA

96. Daisuke Kihara, Purdue University, West Lafayette, USA

97. Ron Kikinis, Harvard University, Harvard Medical School and BWH, USA

98. Hyunsoo Kim, Georgia Institute of Technology, USA

99. Sun Kim, Indiana University, USA

100. Meena Kishore Sakharkar, Nanyang Technological University, Singapore

101. Thomas Knudsen, University of Louisville, USA

102. Shawn Konecni, University of Massachusetts Lowell, USA

103. Stefan C. Kremer, University of Guelph, Canada

104. Rui Kuang, University of Minnesota, USA

105. Vipin Kumar, University of Minnesota, USA

106. Michael Kwok-Po Ng, Hong Kong Baptist University, Hong Kong

107. Walter Land, Jr., Binghamton University, USA

108. Chang-Shing Lee, National University of Taiwan, Taiwan

109. Doheon Lee, KIST, Korea

110. Haim Levkowitz, University of Massachusetts Lowell, USA

111. Christina M. Li, Duke University, USA

112. Guo-Zheng Li, Shanghai University, China

113. Guoliang Li, National University of Singapore, Singapore

114. Jinyan Li, Institute for Infocomm Research, Singapore

115. Lihua Li, Hangzhou Dianzi University, China

116. Yingshu Li, Georgia State University, USA

117. Hongen Liao, University of Tokyo, Japan

118. Li Liao, University of Delaware, USA

119. Lily Liang, University of the District of Columbia, USA

120. Yanchun Liang, Jilin University, China

121. Yulan Liang, University at Buffalo, USA

122. Zhi-Pei Liang, University of Illinois at Urbana-Champaign, USA

123. David Lightfoot, Southern Illinois University, USA

124. Timothy G. Lilburn, ATCC, USA

125. Guohui Lin, University of Alberta, Canada

126. Malinda Lingwall, Indiana University, Bloomington, USA

127. Qingzhong Liu, New Mexico Tech, USA

128. Tianming Liu, Harvard University, USA

129. Wei Liu, Hangzhou Dianzi University, China

130. Ying Liu, University of Texas at Dallas, USA

131. Yunlong Liu, Indiana University School of Medicine, USA

132. Xiaole Shirley Liu, Harvard University, Harvard School of Public Health, USA

133. Gary Livingston, University of Massachusetts Lowell, USA

134. Marco Loog, University of Copenhagen, Denmark

135. Shiyong Lu, Wayne State University, USA

136. Xiaochun Lu, Duke University, USA

137. Xinghua Lu, Medical University of South Carolina, USA

138. Yi Lu, University of Maryland East Shore, USA

139. Dijun Luo, University of Texas at Arlington, USA

140. Tiejian Luo, Chinese Academy of Science, China

141. Zuojie Luo, Guangxi Medical University, China

142. Carol Lushbough, University of South Dakota, USA

143. Yan Ma, Guangxi Medical University, China

144. Anant Madabhushi, Rutgers University, USA

145. Man-Wai Mak, Hong Kong Polytechnic University, Hong Kong

146. Ion Mandoiu, University of Connecticut, USA

147. Ilias Maglogiannis, University of Aegean, Greece

148. Kezhi Mao, Nanyang Technological University, Singapore

149. Marcos Martin-Fernandez, Harvard University, USA

150. Osamu Maruyama, Kyushu University, Japan

151. Kenneth Marx, University of Massachusetts Lowell, USA

152. Vasilis Megalooikonomou, Temple University, USA

153. Linda Molnar, National Cancer Institute, NIH, US Dept. of Health, USA

154. Jason H. Moore, Dartmouth College, Hitchcock Medical Center, USA

155. Shahriar Movafaghi, University of Southern New Hampshire, USA

156. Palaniswami Mprof, Melbourne University, Australia

157. Srinivas Mukkamala, New Mexico Tech, USA

158. Kayvan Najarian, University of North Carolina at Charlotte, USA

159. Giri Narasimhan, Florida International University, USA

160. Jennifer Neary, University of Texas at San Antonio, USA

161. Kathleen Neumann, Western Illinois University, USA

162. See-Kiong Ng, Institute for Infocomm Research, Singapore

163. Jun Ni, University of Iowa, USA

164. Juan J. Nieto, Universidad de Santiago de Compostela, Spain

165. Zoran Obradovic, Temple University, USA

166. Christopher Oldfield, Indiana University – Purdue University, USA

167. William Osborne, Southern Illinois University Carbondale, USA

168. Vasile Palade, Oxford University, UK

169. Haesun Park, Georgia Institute of Technology, USA

170. Minseo Park, University of Massachusetts Lowell, USA

171. Peter Park, Harvard University, USA

172. Srinivasan Parthasarathy, Ohio State University, USA

173. William Perrizo, North Dakota State University, USA

174. Vinhthuy Phan, Univerisity of Memphis, USA

175. Aleksandar Poleksic, University of Northern Iowa, USA

176. David Pollack, University of Colorado Health Science Center, USA

177. Jun Qin, Duke University, USA

178. Yifeng Qin, Guangxi Medical University, China

179. Sridhar Ramachandran, Indiana University Southeast, USA

180. Marco Ramoni, Massachusetts Institute of Technology and Harvard University, USA

181. Michael L. Raymer, Wright State University, USA

182. Arun Rawat, University of Southern Mississippi, USA

183. Chandan Reddy, Wayne State University, USA

184. J. Paul Robinson, Purdue University, USA

185. Usman W. Roshan, New Jersey Institute of Technology, USA

186. Hagit Shatkay, Queen's University, Canada

187. John Sharko, University of Massachusetts Lowell, USA

188. Bingxin Shen, Brookhaven National Lab. – Stony Brook University (SUNY), USA

189. Hong-Bin Shen, Harvard University, USA

190. Li Shen, Indiana University, USA

191. Prawal Sinha, Indian Institute of Technology Kanpur, India

192. Saurabh Sinha, University of Illinois at Urbana-Champaign, USA

193. Scott Smith, Boise State University, USA

194. Craig A. Stewart, Indiana University, Bloomington, USA

195. Yijun Sun, University of Florida, USA

196. Andrew Sung, New Mexico Tech, USA

197. Wing-Kin Sung, National University of Singapore, USA

198. Jack Szostak, Harvard University, USA

199. David Taniar, Monash University, Australia

200. Jijun Tang, University of South Carolina, USA

201. Yuchun Tang, Georgia State University, USA

202. Rahman Tashakkori, Appalachian State University, USA

203. Jeff Thai, University of Illinois, USA

204. My T. Thai, University of Florida, USA

205. Homayoun Valafar, University of South Carolina (Columbia), USA

206. Thanos Vasilakos, University of Western Macedonia, Greece

207. Vladimir N. Uversky, Indiana University School of Medicine, USA

208. Jason T.L. Wang, New Jersey Institute of Technology, USA

209. Jianzhong Wang, Northeast Normal University, China

210. Li-San Wang, University of Pennsylvania, USA

211. Liangjiang Wang, Clemson University and Greenwood Genetic Center, USA

212. Lipo Wang, Nanyang Technological University, Singapore

213. May D. Wang, Georgia Institute of Technology, USA

214. Partrick Wang, Northeastern University, USA

215. Wei Wang, University of North Carolina at Chapel Hill, USA

216. Xiangyun Wang, Amgen Inc., USA

217. Yufeng Wang, University of Texas at San Antonio, USA

218. Yuhang Wang, Southern Methodist University, USA

219. Zhengyuan Wang, Washington University, USA

220. Lonnie R. Welch, Ohio University, USA

221. Limsoon Wong, National University of Singapore, Singapore

222. Stephen Wong, Harvard University, Harvard Medical School and BWH, USA

223. Hongwei Wu, University of Georgia, USA

224. Qishi Wu, University of Memphis, USA

225. Eric P. Xing, Carnegie Mellon University, USA

226. Fei Xiong, Dartmouth College, USA

227. Dong Xu, University of Missouri-Columbia, USA

228. Jinbo Xu, Toyota Technological Institute at Chicago, USA

229. Min Xu, University of Southern California, USA

230. Ying Xu, University of Georgia and Oak Ridge National Lab., USA

231. Changhui Yan, Utah State University, USA

232. Hong Yan, City University of Hong Kong, Hong Kong

233. Xiting Yan, University of Southern California, USA

234. Jie Yang, Shanghai Jiaotong University, China

235. Laurence T. Yang, St. Francis Xavier University, Canada

236. Luge Yang, University of California at Los Angeles, USA

237. Jack Y. Yang, Harvard University, Harvard Medical School, USA

238. Mary Qu Yang, National Human Genome Research Inst., NIH; & Oak Ridge, DOE, USA

239. Mohammed Yeasin, University of Memphis, USA

240. Tulay Yildirim, Yildiz Technical University, Turkey

241. Illhoi Yoo, University of Missouri, USA

242. Jingkai Yu, Wayne State University, USA

243. John Jiazheng Yuan, Southern Illinois University, USA

244. P. Yuan, IBM, USA

245. Feng Yue, University of South Carolina, USA

246. Mehdi Zagerham, Southern Illinois University, USA

247. Mohammed Zaki, Rensselaer Polytechnic Institute, USA

248. Leonid Zaslavsky, NCBI, NLM, National Institutes of Health, USA

249. Alex Zelikovsky, Georiga State University, USA

250. Jianyang Zeng, Duke University, USA

251. Aidong Zhang, University at Buffalo (SUNY), USA

252. Byoung-Tak Zhang, Seoul National University, South Korea

253. Kangyu Zhang, University of Southern California, USA

254. Liqiang Zhang, Indiana University South Bend, USA

255. Meng Zhang, Jilin University, China

256. Michael Q. Zhang, Cold Spring Harbor Laboratory, USA

257. Qing Zhang, Pharmaceutical Industry, USA

258. Yanqing Zhang, Georgia State University, USA

259. Weixiong Zhang, Washington University (St. Louis), USA

260. Xiang-Sun Zhang, Chinese Academy of Sciences, China

261. Mingyuan Zhao, University of Electronic Science and Technology of China, China

262. Ying Zhao, Tsinghua University, China

263. Jim Zheng, Medical University of South Carolina, USA

264. Tian Zheng, Columbia University, USA

265. Wei Zhong, University of South Carolina Upstate, USA

266. Jianping Zhou, University of Massachusetts Lowell, USA

267. Jie Zhou, Northern Illinois University, USA

268. Joe Zhou, Monash University, Australia

269. Joe Jizhong Zhou, Oak Ridge National Lab. DOE and University of Oklahoma, USA

270. Zhi-Hua Zhou, Nanjing University, China

271. Michelle M. Zhu, Oak Ridge National Lab. and Southern Illinois University, USA

272. Hanqi Zhuang, Florida Atlantic University, USA

273. Igor B. Zhulin, Oak Ridge National Lab, DOE and University of Tennessee, USA

### Members of steering and advisory committee

1. Hamid R. Arabnia, University of Georgia, USA

2. Brian D. Athey, University of Michigan and NCIBI, USA

3. Ruzena Bajcsy, University of California, Berkeley, USA

4. Mark Borodovsky, Georgia Institute of Technology, USA

5. Nikolaos G. Bourbakis, Wright State University, USA

6. Philip E. Bourne, University of California at San Diego, USA

7. George T. Y. Chen, Harvard University & Massachusetts General Hospital, USA

8. Yuehui Chen, University of Jinan, China

9. Keith A. Crandall, Brigham Young University, USA

10. Youping Deng, University of Southern Mississippi, USA

11. Laura L. Elnitski, National Human Genome Research Institute, NIH, USA

12. Okan K. Ersoy, Purdue University, West Lafayette, USA

13. Joydeep Ghosh, University of Texas, Austin, USA

14. Georges Grinstein, University of Massachusetts, Lowell, USA

15. Tony Xiaohua Hu, Drexel University, USA

16. Xudong Huang, Harvard University, Harvard Medical School and BWH, USA

17. Eric Jakobsson, University of Illinois at Urbana-Champaign, USA

18. George J. Klir, Binghamton University, USA

19. Thomas Knudsen, University of Louisville, USA

20. Ming Li, University of Waterloo, Canada

21. Zhi-Pei Liang, IEEE/EMBS Vice-President for Conferences, University of Illinois, USA

22. Jun Ni, University of Iowa, USA

23. Andrzej Niemierko, Harvard University & Massachusetts General Hospital, USA

24. Saranummi Niilo, Technical Research Center, Finland

25. Yi Pan, Georgia State University, USA

26. Corrado Priami, University of Trento, Italy

27. John Quackenbush, Harvard School of Public Health and Danna-Faber Cancer Institute, USA

28. Carmelina Ruggiero, University of Genova, Italy

29. Patrick S. Wang, Northeastern University, USA

30. Limsoon Wong, National University of Singapore, Singapore.

31. Jonathan Wren, University of Oklahoma, USA.

32. Jack Y. Yang, Harvard University, Harvard Medical School, USA

33. Mary Qu Yang, National Human Genome Research Inst., NIH; & Oak Ridge, DOE, USA

34. Xin Yao, University of Birmingham, UK

35. Lotfi A. Zadeh, University of California, Berkeley, USA

36. Yanqing Zhang, Georgia State University, USA

37. Michelle M. Zhu, Oak Ridge National Lab, DOE and Southern Illinois University, USA

The IEEE 7th Bioinformatics and Bioengineering committees would like to acknowledge our appreciation of the Institute of Electrical and Electronics Engineers (IEEE), IEEE Systems, Man and Cybernetics Society, IEEE Engineering in Medicine and Biology Society, the International Society of Intelligent Biological Medicine (ISIBM) and the National Science Foundation (NSF) for their academic supports and sponsorships, as well as the support and collaboration from the IEEE Computer Society. The IEEE 7th Bioinformatics and Bioengineering was 100% fully sponsored both financially and academically by the IEEE.

## Meeting website

The home page of the IEEE 7^th ^Bioinformatics and Bioengineering is hosted at the website of the International Society of Biological Medicine. The information about the conference can be found on the web site: http://www.isibm.org/BIBE07

## Conclusion

As upcoming emerging fields, bioinformatics, genomics and personalized medicine integrate science and engineering with modern state-of-art high performance computing and statistical learning techniques. The synergy of bioinformatics, genomics and personalized medicine has been well received by researchers. Each research paper was reviewed and ranked by experts in fields. As a result of the rigorous review process and stringent evaluation, the committee selected 27 papers for this *BMC Genome *supplementary issue. In cooperation with the International Society of Intelligent Biological Medicine http://www.isibm.org and the World Congress on Computer Science, Computer Engineering and Applied Computing http://www.world-academy-of-science.org, the IEEE 7^th ^Bioinformatics and Bioengineering at Harvard Medical School has provided a unique platform and infrastructure to promote interdisciplinary and multidisciplinary research and education with a theme that genomics, molecular imaging, bioinformatics, and bio-nano-info integration are synergistic components of translational medicine and personalized healthcare research. The IEEE flagship conference aimed to promote synergistic inter/multidisciplinary research and education to solve important but difficult biomedical problems that need both expertise in biomedical sciences and engineering. This *BMC Genomics *issue is a product of part of the achievement of the IEEE 7^th ^Bioinformatics and Bioengineering at Harvard Medical School in promoting emerging cutting-edge research fields with most updated research trends, novel technologies, breakthrough ideas and scientific discoveries.

## Competing interests

The authors declare that they have no competing interests.

## Authors' contributions

Jack Y. Yang and Mary Qu Yang wrote the initial version of the paper. Hamid R. Arabnia and Youping Deng finalized the paper. All co-authors have read and unanimously agreed on the paper, participated in the program and scientific review committee to review 600+ submissions and served as co-editors of the *BMC Genomics *supplement. Jack Y. Yang served as the General Chair of IEEE 7^th ^Bioinformatics and Bioengineering and Co-PI of the NSF grant, Mary Qu Yang served as Scientific Review, Advisory and Steering Committee Chair, Hamid R. Arabnia served as Award Committee Chair, NSF Student Travel Fellowship Committee Chair and Program Co-Chair. Youping Deng served as Financial Chair, Program Co-Chair and Co-PI of the NSF grant.
